# Endogenous siRNAs and piRNAs derived from transposable elements and genes in the malaria vector mosquito *Anopheles gambiae*

**DOI:** 10.1186/s12864-015-1436-1

**Published:** 2015-04-10

**Authors:** Inna Biryukova, Tao Ye

**Affiliations:** Department of Vector Biology, Max Planck Institute for Infection Biology (MPIIB), Berlin, 10117 Germany; Microarrays and deep sequencing platform, Institut de Génétique et de Biologie Moléculaire et Cellulaire (IGBMC), Illkirch, Cedex 67404 France

**Keywords:** endo-siRNAs, endo-piRNAs, *Anopheles gambiae*, Transposable elements

## Abstract

**Background:**

The siRNA and piRNA pathways have been shown in insects to be essential for regulation of gene expression and defence against exogenous and endogenous genetic elements (viruses and transposable elements). The vast majority of endogenous small RNAs produced by the siRNA and piRNA pathways originate from repetitive or transposable elements (TE). In *D. melanogaster*, TE-derived endogenous siRNAs and piRNAs are involved in genome surveillance and maintenance of genome integrity. In the medically relevant malaria mosquito *Anopheles gambiae* TEs constitute 12-16% of the genome size. Genetic variations induced by TE activities are known to shape the genome landscape and to alter the fitness in *An. gambiae*.

**Results:**

Here, using bioinformatics approaches we analyzed the small RNA data sets from 6 libraries formally reported in a previous study and examined the expression of the mixed germline/somatic siRNAs and piRNAs produced in adult *An. gambiae* females. We characterized a large population of TE-derived endogenous siRNAs and piRNAs, which constitutes 56-60% of the total siRNA and piRNA reads in the analysed libraries. Moreover, we identified a number of protein coding genes producing gene-specific siRNAs and piRNAs that were generally expressed at much lower levels than the TE-associated small RNAs. Detailed sequence analysis revealed that *An. gambiae* piRNAs were produced by both “ping-pong” dependent (TE-associated piRNAs) and independent mechanisms (genic piRNAs). Similarly to *D. melanogaster,* more than 90% of the detected piRNAs were produced from TE-associated clusters in *An. gambiae.* We also found that biotic stress as blood feeding and infection with *Plasmodium* parasite, the etiological agent of malaria, modulated the expression levels of the endogenous siRNAs and piRNAs in *An. gambiae*.

**Conclusions:**

We identified a large and diverse set of the endogenously derived siRNAs and piRNAs that share common and distinct aspects of small RNA expression across insect species, and inferred their impact on TE and gene activity in *An. gambiae*. The TE-specific small RNAs produced by both the siRNA and piRNA pathways represent an important aspect of genome stability and genetic variation, which might have a strong impact on the evolution of the genome and vector competence in the malaria mosquitoes.

**Electronic supplementary material:**

The online version of this article (doi:10.1186/s12864-015-1436-1) contains supplementary material, which is available to authorized users.

## Background

Small non-coding RNAs (ncRNAs) are involved in regulation of gene expression, RNA based immunity and activity of transposable elements (TEs) and their remnants. In *D. melanogaster* two small RNA silencing pathways, small interfering RNAs (siRNA) and piRNA (PIWI-interacting RNAs), regulate gene expression by silencing specific genes and TEs both in germline and soma [1-7]. In *Dipteran* insects siRNAs represent a population of small RNA 21-nucleotides (nt) in length. siRNA is originated from exo- and endogenous double stranded RNA (dsRNA), which is processed by the RNAse III enzyme Dicer-2 (Dcr-2) into 21-nt siRNA duplexes. Once produced and loaded into RNA-induced silencing complex (RISC), siRNAs guide in sequence-specific manner the recognition and cleavage of the target RNA molecules by the Argounate-2 (Ago-2) containing RISC. Similarly to *D. melanosgaster*, the Ago-2 expression in mosquitoes is required for antiviral defence. Depletion of Ago-2 in *An. gambiae* and *Ae. aegypti* mosquitoes respectively infected with *O’nyong-yong* virus and Sindbis virus resulted in increased virus replication [8,9].

piRNAs represent a population of small RNAs ranging from 24 to 30-nt in length, that are specifically expressed in gonads of metazoans and provide genome integrity over generations [10-12]. In insects the major source of piRNAs is transposable genetic elements that are considered as selfish or parasitic elements of the host genome. piRNAs post-transcriptionally silence TE expression through the piRNA-guided cleavage of the transposon mRNA [13]. In addition, piRNAs can mediate transcriptional control of TE activity [6]. The piRNA-mediated RNA silencing requires an association with Ago family members Ago-3, Aubergine (Aub) and PIWI (P-element induced wiped testis) that operate at the heart of the piRNA silencing pathway [13-15]. In contrast to siRNAs, piRNAs are produced in Dicer-independent manner from a long single-stranded RNA transcribed from repetitive elements or genomic loci known as piRNA clusters. This transcript is processed into primary piRNAs that are usually antisense to TE transcript. These primary piRNAs direct to cleave sense TE transcripts and initiate a reciprocal amplification cycle so-called “ping-pong” amplification loop [13,15]. PIWI-class members exhibit strand specific interaction with piRNAs, PIWI and Aub are associated with antisense TE-piRNAs; while Ago-3 is associated with sense TE-piRNAs [13,15]. The hallmarks of the ping-pong mediated amplification of piRNAs are strong U1 bias for Aub-associated piRNAs and A10 of Ago-3-associted piRNAs. In flies, Aub and Ago-3 are not expressed in the ovarian somatic sheet (OSS) cell line and as a result primary piRNAs lack a ping-pong signature, yet showing strong bias for 5′ U [16]. In addition to the function of piRNAs in controlling TE activity, the piRNA pathway is involved in antiviral response in *D. melanogaster* and mosquito cell lines [4,17-22]. Altogether, the piRNA-mediated pathway constitutes an adaptive immune response that recognizes and silences invading parasitic genetic elements [15,23].

TEs represent a large part of dispersed repetitive elements, recognized as one of the major cause of intra-genomic variation and genome diversification. The vertically transmitted TEs can disperse rapidly into populations [24]. *De novo* TE remobilization and insertion are capable of significantly influencing genome stability, host gene transcription, splicing or RNA editing. Subsequent TE invasion, dispersion, inactivation and deterioration forms a unique genome landscape and represents a process of “molecular domestication” of mobile elements [25]. TEs spread over genome via an RNA or a DNA intermediate and have been classified accordingly into two classes, class I (retrotransposons) and class II (DNA transposons). Class I is composed of long terminal repeat (LTR) retrotransposons and non-LTR (NLTR) retrotransposons that are structurally similar to retroviruses and require a reverse transcription step for retrotransposition. Class II TEs includes DNA transposons with i) cut-and-paste mechanism of transposition, ii) rolling-circle DNA transposons (Helitrons) and iii) self-synthesizing DNA transposons (Polintrons) [26]. In *An. gambiae,* TEs constitute about 12-16% of the euchromatic regions and more than 60% of the heterochromatic regions of the genome [27,28]. Overall more than 350 different transposon families have been identified in the genome of *An. gambiae,* the most abundant are LTR-retrotransposons, short interspersed nuclear elements (SINEs) and miniature inverted transposable elements (MITEs) superfamilies [24,27,29].

It has been reported that hematophagous arthropod vector mosquitoes produce virus-derived small RNAs in the soma in response to viral infection [19,20,30,31]. Furthermore, these mosquitoes produce endogenous siRNAs and piRNAs that map to TEs and protein coding genes [31,32]. The precise function of endogenously produced siRNAs and piRNAs remains largely unknown in mosquito species. Both the siRNA and piRNA pathways represent an important aspect of genetic variation, which might have a strong impact on evolution of the host genome landscape and influence the fitness and vector competence in the malaria mosquito. Using previously reported deep sequenced small RNA libraries [33] we examined the expression of endogenous siRNAs and piRNAs produced in adult *An. gambiae* females. We identified a large set of TE- and gene-associated siRNAs and piRNAs, which might be involved in the regulation of transposable element activity and gene expression in soma and germline in *An. gambiae*. Detailed sequence analysis revealed that *An. gambiae* piRNAs were produced by both “ping-pong” dependent and “ping-pong” independent mechanisms. We also found that regular and infectious blood feeding modulated the expression levels of the endogenous small RNA populations and the core components of the siRNA and piRNA pathways in *An. gambiae.*

## Results

### Small RNA sequencing

**Figure 1:**
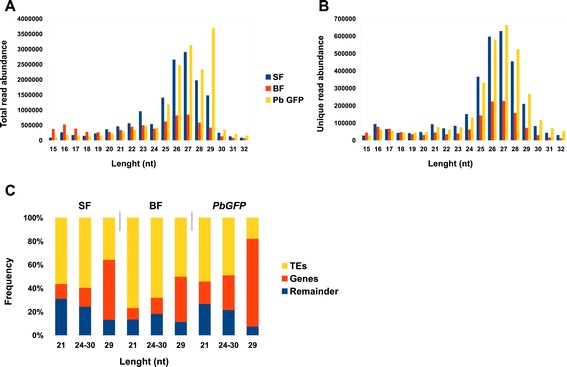
**Sequencing summary of siRNA and piRNA populations in** ***An. gambiae*** **.** Size distribution for the total number of sequence reads **(A)** and for unique sequence reads **(B)** in *An. gambiae* cDNA libraries derived from sugar (SF), blood-fed (BF) and *P. berghei* infected (*PbGFP*) mosquitoes. The cDNA libraries from replicate were collapsed; the length and abundance of small RNA reads in *An. gambiae* cDNA libraries are as indicated. **(C)** Read frequency for siRNAs (peaking at 21 nt) and piRNAs (peaking at ~24-30 nt) mapped to repetitive elements, consisting largely of TEs, coding genes and other sequences (remainder) in *An. gambiae* cDNA libraries.

**Figure 2:**
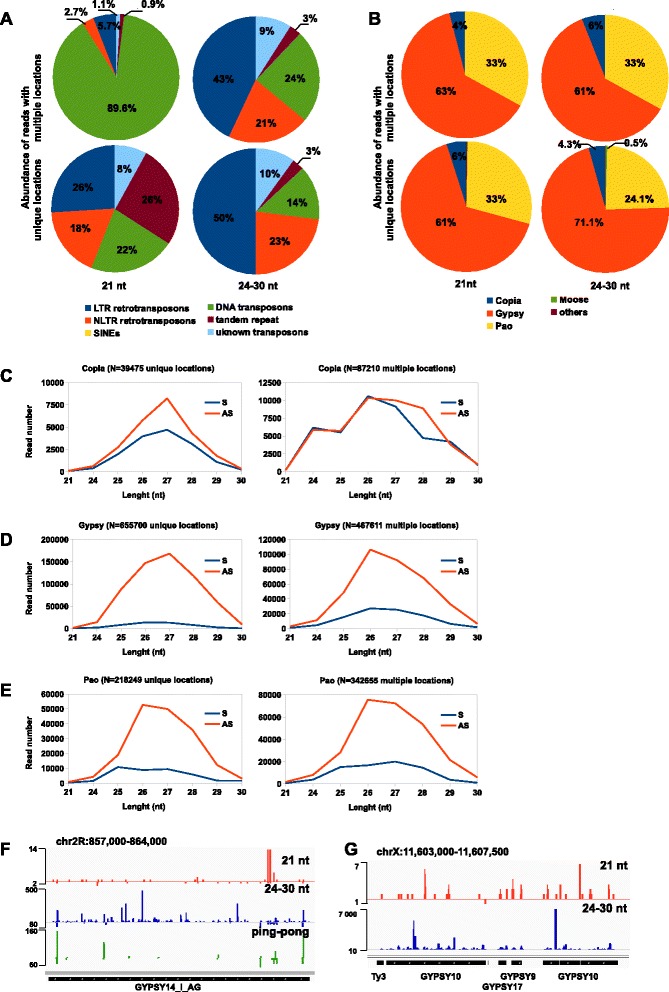
**TE-piRNAs and TE-siRNAs in** ***An. gambiae*** **. (A)** Read frequency for a siRNA population (21 nt) and a piRNA population (24-30 nt) associated with various classes of TEs in *An. gambiae* (EnsemblMetazoa). **(B)** Read frequency for piRNAs mapped to class I superfamilies (LTR and NLTR TEs). **(C-E)** Small RNA read frequency and distribution mapped to various families of TEs. Distribution of reads uniquely mapped to *An. gambiae* genome (left) and reads mapped to the genome five and more times (right). N indicates the number of reads; sense reads (S) and antisense reads (AS) are shown as indicated. **(F-G)** Genomic profile of the 21-nt (red), 24-30-nt (blue) and piRNA “ping-pong” paired (green) reads mapped to the sense and antisense strand of *gypsy* transposon (represented by an abundantly expressed single full-length copy **(F)** and a cluster of truncated and Solo-LTR copies **(G)**) in the *An. gambiae* genome.

**Figure 3:**
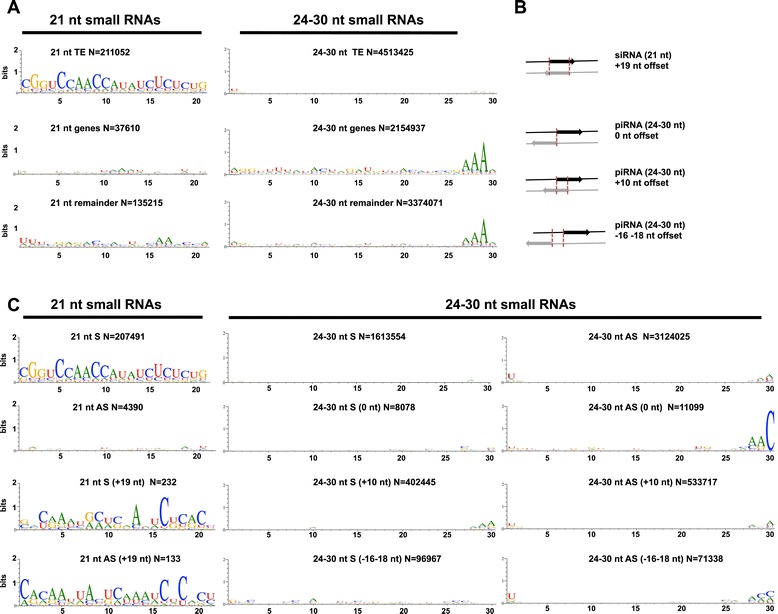
**Sequence properties of TE-piRNAs and TE-siRNAs in** ***An. gambiae*** **. (A)** Sequence compilations showing relative nucleotide frequency per position for the siRNA population (21 nt) and the piRNA population (24-30 nt) associated with TEs (EnsemblMetazoa), coding genes and remaining sequences in *An. gambiae* (VectorBase). In all graphs, N indicates the number of reads. **(B)** Schematic representation of overlapping sequence offsets for siRNA and piRNA pairs. **(C)** Nucleotide composition of all TE-derived reads of length 21-nt (siRNAs) and 24-30-nt (piRNAs) mapped to the sense and antisense strand of the *An. gambiae* TE dataset. The overlap analysis offsets of siRNAs and piRNAs, sense reads and antisense reads are shown as indicated.

We performed analysis of small RNA populations recovered from the deep sequenced small RNA libraries published in [33] to determine i) diversity of the mixed germline/somatic endogenous siRNAs and piRNAs in *An. gambiae* adult females ii) how endogenous siRNAs and piRNAs respond to blood feeding and *Plasmodium berghei* infection. We used a combined strategy [16,32,34-36] following the annotation of piRNAs and siRNAs by analysing sequence signature characteristics of piRNAs/siRNAs and performing a validation by a homology-based analysis of the identified sequences to known piRNA and siRNA loci in *D. melanogaster* and *Ae. aegypti*. We filtered out the known miRNAs, rRNAs, tRNAs and snoRNAs (miRBase, VectorBase, Rfam) from the libraries for further analysis. The reference strain, PEST (AgamP3.8, VectorBase), which segregates into two different haplotypes (S and M) for certain regions of the genome [27] was used to map small RNA reads to the *An. gambiae* genome. The analysed genome of the susceptible *An. gambiae* G3 strain (S haplotype) is divergent from the PEST genome, therefore we allowed 2-nt mismatching for mapping. The analysis of the small RNA populations ranging from 15 to 44-nt in length from sugar, blood and *P. berghei*-infected mosquito libraries, revealed that sequences of 20-23-nt lengths (a 21-nt peak length) and 24-30-nt (a 27-nt peak length) were predominantly recovered (Figure 1A-B; Additional file 1: Table S1). Sequences in the 21-nt and 24-30-nt size range, not annotated as a previously known non-coding RNAs, and passing the cut-off at least 10 read uniquely mapped per individual endo-siRNA or endo-piRNA were classified accordingly as candidate siRNAs and piRNAs. Additionally to the 21-nt and 27-nt peak lengths we also detected a large number of other small non-coding RNAs peaking at 16 nt and 23 nt (Figure 1A-B, Additional file 2: Table S2 and Additional file 3: Table S3). Analysis of the *An. gambiae* genome complexity revealed that protein coding genes constitute 7% of the genome, TEs – 12-16% and total intergenic regions ~78% [27]. We found the following distribution of 21-nt reads in sugar-fed mosquito libraries, 12.6% mapped to the protein coding genes, 31% mapped to intergenic regions and 56.4% mapped to repetitive and transposable elements (Figure 1C; Additional file 2: Tables S2 and Additional file 3: Tables S3). The 24-30-nt read distribution showed a similar tendency mapping to protein coding genes (16%) and intergenic regions (24%); 60% were originated from repetitive elements and TEs (Figure 1C; Additional file 2: Tables S2 and Additional file 3: Tables S3). Moreover, we observed a similar frequency for genic 21-nt and 24-30-nt reads in mosquitoes after regular blood feeding (Figure 1C), whereas TE-associated number of reads was increased relatively to the levels in sugar-fed females. Interestingly, we also noticed higher density of the TE-associated and genic 21-nt and 24-30-nt reads in mosquitoes after infectious blood feeding (Figure 1C). Overall, in our libraries more than 55% of all 21-nt and 24-30-nt reads mapped to known TEs (Figure 2A,B). Importantly, TE-associated 24-30-nt reads exhibited a strong bias towards the antisense strand recapitulating the sequence signatures of piRNAs produced via ping-pong dependent piRNA pathway (Figures 2 and 3). A similar pattern for piRNAs has been previously reported for *D. melanogaster* and *Ae. aegypti* [15,32]. Furthermore, some *An. gambiae* endo-piRNAs matched to known *Ae. aegypti* piRNAs [20,32] indicating identification of *bona fide* piRNAs.

### TE-specific siRNA and piRNA expression in *An. gambiae*

Repetitive and transposable elements represent the major source of endogenous piRNAs and siRNAs in *Dipteran* insects and other animals [13,15,37-40]. The genome landscape of *An. gambiae* is represented by members of both classes, class I (retrotransposons) and class II (DNA transposons) [24,27,29], which constitute 6.85% and 5.9% respectively of the total *An. gambiae* genome size [27,32,41]. We analysed genomic distribution and density of endo-siRNAs (21-nt) and endo-piRNAs (24-30-nt) sequence reads mapped to the collection of repetitive and mobile elements from the genome of *An. gambiae* using EnsemblMetazoa database in sugar-fed mosquitoes (Figure 2A-E). Mapping of 21-nt reads to the class I and class II of TEs revealed that 10.4% and 86% of total endo-siRNA reads were associated with retrotransposons and DNA transposons respectively. Besides, ~67% of the total endo-piRNA reads (24-30-nt) were derived from retrotransposons and 21% from DNA transposons. The distribution of the small RNA reads derived from unique genomic loci was consistent with the distribution of the multiply mapped 24-30-nt reads (Figure 2A); moreover, the uniquely mapped 21-nt reads showed a redistribution of read frequencies (26%, 18%, 22% for the LTR, NLTR retrotransposons and DNA transposons, respectively) compared with the total and multiply mapped 21-nt reads (Figure 2A).

Recent analysis of the TE distribution revealed that class I constitutes two-third and class II - one-third of the total TEs identified in the genome of *An. gambiae* (34%, 33%, 33% for the LTR, NLTR retrotransposons and DNA transposons, respectively) [24]. Therefore, we further analysed in detail the class I LTR and NLTR-derived siRNAs and piRNAs (Figure 2B-G). The class-I LTR-TEs are represented by three retrotransposon superfamilies: *copia* (≥800 copies), *Pao-Bel* (~1000 copies) and *gypsy* (≥1000 copies) [27]. They constitute approximately 4%, 35% and 52% respectively of the total TE number in the genome of *An. gambiae* [29]. The distribution of the total 24-30-nt read frequency revealed that 5.6% of TE-piRNAs were associated with *copia*, 30% with *Pao-Bel* and 64% with *gypsy*, indicating that *copia*, *Pao-Bel* and *gypsy* were likely the most actively transcribed LTR-retrotransposons in our libraries. Next, we analysed the density and distribution of uniquely and multiply mapped 21-nt and 24-30-nt reads derived from these LTR-TEs (Figure 2C-E; Additional file 4: Figure S1). The pool of multiply mapped 21-nt and 24-30-nt (five genomic location and more) were considered respectively as siRNAs and piRNAs derived from dispersed copies of the LTR-TEs. We found that *copia* produced uniquely mapped siRNAs and piRNAs with a bias towards antisense orientation (Figure 2C). Besides, among multiply mapped piRNAs, only class of 27-28-nt reads exhibited a mild antisense bias. *gypsy* and *Pao-Bel* generated a high proportion of uniquely and multiply mapped piRNA reads with a strong antisense bias (Figure 2D-E). In *D. melanogaster gypsy* produced abundantly both classes of TE-associated small RNAs, siRNAs and piRNAs [16]. *An. gambiae gypsy* abundantly produced piRNAs, predominantly 26-27-nt reads in length and a very low number of siRNAs unlike *Drosophila gypsy* (Figure 2D,F-G). *gypsy* elements represent the most abundant and diverse LTR superfamily in the *An. gambiae* genome [24,41]. In total, 42% and 15% of *gypsy* copies represent remnants of the *gypsy* full-length transposon, so-called solitaire Solo-LTR and truncated copies respectively [24]. The sense and antisense distribution of 21-nt and 24-30-nt reads along the sequence of *gypsy* revealed a multiple hot spots of *gypsy*-specific siRNAs and piRNAs that were spread along the TE sequence and LTRrs (Figure 2F-G).

Class-I NLTR-retrotransposons is composed of 14 superfamilies in the *An. gambiae* genome [24,42]. The NLTRs represent the most abundant and diverse type of TEs in *An. gambiae* [24]. We noticed that the total abundance of NLTR-piRNAs and siRNAs was low relative to its size (Figure 2A,B) probably due to extremely low proportion of full-length elements in NLTRs in comparison to LTRs [24]. *Jockey*, *CR1*, *RTE* and *Outcast* elements represent the most abundant superfamilies (15%, 63%, 11% and 7% respectively of the total NLTR-TEs in the genome of *An. gambiae*; [29]). We found that 44% of the total NLTR-piRNA reads were derived from *Jockey*, *CR1*, *RTE* and *Outcast* retrotransposons in the analysed small RNA libraries (Additional file 2: Table S2). We also observed that the *SINEX-1* family, which represents a large part of the total NLTR-TEs [24] was associated with a low number of siRNA and piRNA reads in our small RNA libraries (Additional file 4: Figure S1).

In the *An. gambiae* genome, DNA transposons are represented by most heterogeneous sets of heavily deteriorated sequences or their remnants in the analysed class II families. Overall, the MITE-like elements, which are lacking any coding capacity, represent 60% of the class II transposons [24]. The most abundant class II-superfamilies are *Tc1-Mariner* (35%), *P* element (10%), *hAT* (8%), *Harbinger* (4%) and novel unknown elements ~32% [29]. In our small RNA libraries, members of *Harbinger*, *Tc1-Mariner, P* element, *Gambol* elements and unknown *TE104184* transposon were the major sources of 21-nt reads and 24-30-nt reads derived from class II DNA transposons (Additional file 4: Figure S1).

Overall, we observed that transcriptional activities of the class I-derived siRNAs and piRNAs correlate with the abundance of the LTR- and NLTR-retrotransposons (*gypsy*, *Pao-Bel*, *copia*; *CR1*, *Jockey*, *Outcast* and *RTE*) in our small RNA libraries (Figure 2A-B, Additional file 4: Figure S1). We also noticed a similar tendency for the class II-derived siRNAs (mapped to *Harbinger*, *Tc-1/Mariner*, *P* element and *Gambol* superfamily). Besides, the total abundance of the DNA transposon-derived piRNAs was low relative to the size of class II DNA transposons in the *An. gambiae* genome.

Next we analysed the relative nucleotide frequencies in the *An. gambiae* TE-siRNAs. The sequence analysis revealed a bias for 5′-end C at position 1 in the total 21-nt reads and in the 21-nt reads that mapped to sense strand of TEs (Figure 3A,C). A similar bias was observed in *D. melanogaster* endogenous siRNAs that often began with C [40]. In addition, we noticed a bias for 5′ U at position 1 for 21-nt reads derived from antisense TE strand (Figure 3A,C). The nucleotide composition across individual *D. melanogaster* TE families showed 5′ U bias in 21-nt reads mapped to antisense strand and no bias in 21-nt reads mapped to sense strand [16,40], this is consistent with the 5′ U preference for the 21-nt antisense reads mapped to specific TEs in *An. gambiae* (results not shown). Next we performed an overlap analysis of sense-antisense 21-nt paired reads. We used (+19-nt) offset to detect 21-nt siRNA duplex with the 3′-end 2-nt overhangs produced by RNAse III enzyme Dcr-2 (Figure 3B). The nucleotide composition analysis revealed a bias at position 1 for 5′ C/G in sense and 5′ C in antisense strand (Figure 3C). In *D. melanogaster* TEs produce siRNAs in the almost equivalent ration between sense and antisense orientation with a slight antisense bias [16]. Unlike in *Drosophila*, in our small RNA datasets the *An. gambiae* TE-siRNAs showed a strong sense bias. The bias was introduced by the 21-nt reads mapped to unknown *TE104184* transposon (Additional file 4: Figure S1), which produced the vast majority of 21-nt sense reads in our libraries. *TE104184* belongs to class II DNA transposons (EnsemblMetazoa) and is represented by more than 800 copies in the *An. gambiae* genome. Non-random distribution and precision of the 5′- and 3′-ends of the *TE104184*-derived 21-nt reads ruled out the possibility that these reads might represent the incidental degradation fragments (Additional file 4: Figure S1).

In contrast to the *An. gambiae* TE-siRNAs, the TE-piRNAs exhibited a strong over-presentation of antisense reads with 5′ U bias (Figure 3C). We analysed in details the occurrence of sense-antisense piRNA arrangements and their nucleotide composition for i) non-paired neighbour piRNAs, ii) overlapped piRNA ping-pong pairs and iii) overlapped piRNA ping-pong pairs with phased arrangement considering a plausible under-representation in our small RNA libraries using 0-nt; +10-nt and (-16-18-nt) offsets respectively ([16], Figure 3B). There was a 5′ U bias for antisense TE-piRNA reads in all analysed offsets and a strong bias for A at position 10 for reads derived from sense strand in +10-nt and (-16-18-nt) offsets (Figure 3B). These results implied that vast majority of the TE-specific piRNAs exhibited the classical hallmarks of ping-pong amplification and most likely were produced by the ping-pong dependent mechanism in *An. gambiae*.

Given the functional association of the core factors of the siRNA pathway (*Dcr-2* and *Ago-2*) and piRNA pathway (*PIWI*-class transcripts *PIWI*, *Aub* and *Ago-3*) in TE silencing, we analysed expression of the most abundant LTR-retrotransposon, *gypsy* and NLTR-retrotransposon, *CR1* in RNA silenced adult female mosquitoes (Additional file 5: Figure S2). The *An. gambiae* PIWI family genes *Ago-3* (*AGAP008862*), *PIWI2/Aub* (*AGAP009509*) and *PIWI1/Aub* (*AGAP011204*) were identified by sequence similarity with the *D. melanogaster Ago* homologs [43,44]. RNAi directed against *Dcr-2*/*Ago-2* and *PIWI*-class genes reduced their transcript levels approximately ~30-50% according to the qPCR-based measurement (Additional file 5: Figure S2). We observed consistent mild de-silencing of *CR1* in *PIWI1-Aub* silenced mosquitoes*,* indicating RNAi-dependent degradation. Besides, expression of the *gypsy* element was not substantially affected in RNA silenced mosquitoes and was varied significantly between biological replicates (Additional file 5: Figure S2) probably due to variegating expression of numerous truncated copies as a result of some subtle differences in mosquito rearing condition.

### Genic siRNA and piRNA loci in the genome of *An. gambiae*

**Figure 4:**
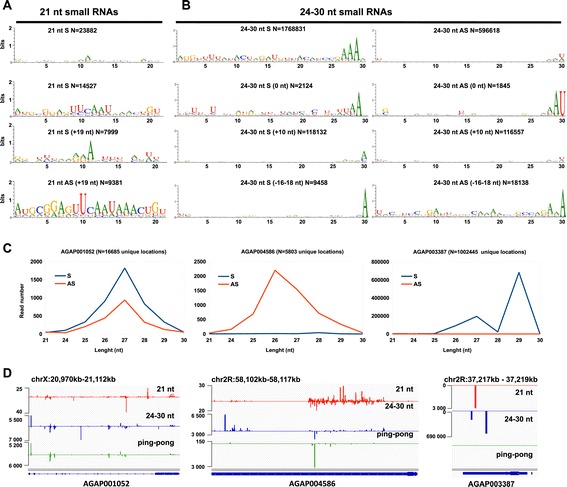
**Sequence characterization of gene-specific piRNAs and siRNAs in** ***An. gambiae*** **.** Sequence properties of sense and antisense siRNA **(A)** and piRNA **(B)** populations mapped to protein coding genes in *An. gambiae* (VectorBase). The offsets for siRNA and piRNA pairs are shown in (Figure 3B). N indicates the number of reads. **(C)** Distribution of unique matching reads mapped to *AGAP001052, AGAP004586* and *AGAP003387* (as representative hot spots of 21-nt and 24-30-nt reads); sense reads (blue); antisense reads (red). **(D)** Genomic profiles showing density of the 21-nt (red), 24-30-nt (blue) and piRNA “ping-pong” paired (green) reads mapped to the sense and antisense strand of *AGAP001052, AGAP004586* and *AGAP003387.*

Given that 9-29% of 21-nt and 24-30-nt reads mapped to protein coding genes in our small RNA libraries (Figure 1C; Additional file 3: Tables S3), we analysed the association between protein coding genes and small RNA sequences (Figure 4) in order to better understand the role of endo-piRNAs and endo-siRNAs. Distribution of the total 21-nt and 24-30-nt reads that mapped to coding genes showed a sense bias for genic piRNAs and siRNAs (Figure 4A-B). The overlap analysis of siRNA pairs revealed almost equivalent sense and antisense distribution in 21-nt reads with a mild antisense bias for (+19 nt) offset. The 24-30-nt piRNA paired reads showed a slight sense bias in the 0 nt and +10 nt offsets, whereas antisense bias (almost two-fold) was detected for (-16-18 nt) offset piRNA pairs (Figure 4B). In addition, we observed consistently 5′ A preference for antisense 21-nt reads and 5′ U for antisense 24-30-nt reads. We also noticed presence of A at position 10 for total 24-30-nt reads in sense orientation (Figure 4B). However, this signature was missing in the (+10 nt) offset paired sense reads, suggesting a ping-pong independent mode of piRNA biogenesis for piRNAs produced by coding genes. Importantly, we did not find in the siRNA pool derived from the protein coding gene any “ping-pong” like signatures meaning that they do not belong to a subset of the piRNA-like small RNAs. It is worth noting that we also observed a production of endogenous piRNAs and siRNAs from intergenic regions in *An. gambiae* genome (Figure 3A). The intergenic 21-nt and 24-30 nt reads also showed a strong 5′ U and 5′ U/A bias respectively (Figure 3A).

Next we analysed the frequency and distribution of the most abundant 21-nt reads and 24-30-nt reads mapped to specific *An. gambiae* genes (Figure 4C-D; Additional file 4: Figure S1). The analysis of small RNA density revealed that two genes *AGAP006442* and *AGAP003387* produced one-third of total 21-nt reads in our libraries (Additional file 3: Tables S2 and Additional file 4: Tables S3; Additional file 4: Figure S1). The *AGAP006442-*associated 21-nt reads mapped to both sense and antisense strands (Additional file 4: Figure S1)*,* whereas *AGAP003387*-derived 21-nt reads were predominantly in sense orientation. Among the top abundant small RNA producing genes, *AGAP001052* produced 21-nt and 24-30-nt reads in almost equivalent proportion from both strands with a sense bias (Figure 4C,D); whereas *AGAP004586-*associated 21-nt reads and 24-30-nt reads showed a strong antisense bias (Figure 4C,D). Both *AGAP001052* and *AGAP004586* were producing paired 24-30-nt reads with “ping-pong” signatures (Figure 4D). The most abundant genic source of 21-nt and 24-30-nt reads in our libraries was *AGAP003387,* which encodes a protein with unknown function and has a single ortholog in *A. quadriannulatus* - *AQUA010285,* putative lipoprotein (VectorBase). Interestingly more than 50% of 24-30-nt reads in sugar- and blood-fed mosquitoes and more than 75% in *P. berghei-*infected mosquitoes were associated with *AGAP003387* (Additional file 3: Table S3). This gene produced small RNA reads predominantly in a sense orientation including two classes of the most abundant reads, 27-nt and 29-nt in length (Figure 4C). Genomic profiling of 21-nt and 24-30-nt reads that mapped to *AGAP003387* showed asymmetric distribution with the highest read density at the 3′ end (Figure 4D). Importantly, the ping-pong piRNA pairs were not detected within the *AGAP003387-*derived population of 24-30-nt reads (Figure 4D). The 3′ end piRNA profile associated with *AGAP003387* might be similar to the profile of genes producing piRNAs from their 3′ UTR in *D. melanogaster* [36,45,46].

The vast majority of endogenous siRNAs and piRNAs originate from transcription of TEs, *cis*-natural antisense transcripts (*cis*-NAT) or long inverted repeat transcripts (structured hairpin RNAs). The genomic organization of *AGAP006442* and *AGAP003387,* which produced the most abundant genic siRNAs, does not show any 3′-end transcript overlapping (VectorBase). Similarly to those, *AGAP004586* is a stand-alone gene, no other known RNA transcripts within a 10 kb genomic window has been annotated (VectorBase), yet we cannot rule out the existence of unknown *cis*-NAT transcripts in the analysed genomic region. *AGAP001052* exhibits convergent arrangement with its neighbour gene showing tail-to-tail organization with *AGAP001051.* The most abundant *An. gambiae* endo-siRNAs were not associated with convergent transcripts, similarly to *Ae. aegypti*, which produced the majority of endo-siRNAs from non-overlapping regions (50%) and only 3.8% from tail-to-tail adjacent transcripts [31]. Interestingly, the detailed examination of the *An. gambiae* loci producing the most abundant classes of genic small RNA reads revealed that 80% of the analysed protein coding genes were framed by nested or clustered repetitive elements or TEs in the surrounding distal and proximal regions.

We also analysed the effect of RNA silencing of *PIWI*-class and *Dcr-2/Ago-2* genes on the *AGAP003387* and *AGAP001052* transcript levels using qPCR. The expression level of *AGAP001052* mRNA was not associated with a significant desilencing; whereas, level of *AGAP003387* transcript was increased in *PIWI-*class silenced mosquitoes (Additional file 5: Figure S2). Correlation analysis of the genic 3′ UTR piRNAs revealed that highly expressed transcripts tend to generate more piRNAs [36]. Curiously, the increased level of *AGAP003387* transcript in blood-fed and infected mosquitoes did not correlate with piRNA levels in our libraries measured at 3 h after feeding (Additional file 3: Table S3, Additional file 6: Figure S3). However, the transcriptional level of *AGAP003387* detected 24 h after infectious blood feeding was significantly lower than after a regular blood feeding (Additional file 6: Figure S3). This might reflect the delayed kinetics of the piRNA-mediated silencing.

Multiple negative feedbacks regulating the core components of small RNA silencing pathways have been reported [1,31,32,36,40]. To identify siRNAs and piRNAs involved in negative feedback circuits in the siRNA and piRNA networks in *An. gambiae*, we analysed 21-nt and 24-30-nt reads that mapped to the main components of the siRNA and piRNA pathways (Table 1). We noticed that the core components of the siRNA biogenesis *Dcr-2,* its cofactors *R2D2* and *Loqs-2* were associated with extremely low read numbers (10 and less reads) mapped to the sense strand. We also observed a similar tendency for the RNA helicase *Spindle E* (*spnE*), DEAD-box helicase DDX17 (*Rm62*) and *Rm68,* which are known to be implicated in RNA biogenesis, microRNA and viral RNA processing in *Drosophila* [47,48]. In the *An. gambiae* genome four putative *Rm62* orthologs were predicted by OrthoDB; among them only *AGAP003663* was associated with a substantial number of 24-30-nt reads mapped in a sense orientation (Table 1). Interestingly, the core components of the microRNA pathway, which produces endogenous small RNAs involved in repression of partially complementary mRNAs, *Drosha, Pasha* and *Dcr-1* were not enriched for 21-nt or 24-30-nt reads (Table 1). Besides two main effectors of the RISC, *Ago-2* (siRISC) and *Ago-1* (miRISC) were associated with higher density of 21-nt and 24-30-nt reads in a sense orientation*.*

**Table 1:** **Genic siRNAs and piRNAs associated with the core components of the small RNA silencing pathways in** ***An. gambiae***

**Small RNA pathway**			**siRNAs**	**piRNAs**
			**21-nt read number**	**24-30-nt read number**
**siRNA**	***Loqs-2***	*AGAP009781*	0	0
	***R2D2***	*AGAP009887*	2	3
	***Dcr-2***	*AGAP012289*	3	8
	***Ago-2***	*AGAP011537*	84	251
**piRNA**	***Ago-3***	*AGAP008862*	0	18
	***Aub-PIWI2***	*AGAP009509*	2	61
	***Aub-PIWI1***	*AGAP011204*	9	93
	***Zucchini***	*AGAP006233*	1	0
	***Armitage***	*AGAP006939*	12	44
	***Tudor***	*AGAP005672*	20	391
	***Vreteno***	*AGAP010722*	1	0
	***Shutdown***	*AGAP011458*	0	19
	***Pimet/HEN1***	*AGAP005646*	0	15
	***tj***	*AGAP010030*	1	8
	***mael***	*AGAP002022*	2	3
	***spnE***	*AGAP002829*	11	9
	***Rm62 (DDX-17)***	*AGAP003663*	29	773
		*AGAP004912*	1	6
		*AGAP005652*	7	93
		*AGAP012045*	14	104
**miRNA**	***Dcr-1***	*AGAP002836*	1	5
	***Drosha***	*AGAP008087*	0	0
	***Pasha***	*AGAP002554*	1	2
	***Ago-1***	*AGAP011717*	122	412

In addition, the core components of the piRNA biogenesis were not associated with genic siRNAs and piRNAs in our libraries (Table 1). The exception was *AGAP005672,* one of the *Tudor*-domain containing orthologues that are known to be involved in the regulation of various small RNAs. Two genes, *traffic jam (tj)* and *maelstrom (mael)*, have been reported to be implicated in the piRNA biogenesis and piRNA production in *D. melanogaster* and in *Ae. aegypti* [32,45]. Unlike *Ae. aegypti* and *D. melanogaster,* the *An. gambiae* orthologs of *tj* and *mael* showed extremely low frequency for the associated genic 21-nt and 24-30-nt reads. Overall, we observed that in adult *An. gambiae* mosquitoes i) genic-siRNAs were expressed at much lower levels than TE-siRNAs in all analysed libraries, ii) genic piRNAs were generally lacking ping-pong sequence signatures and most likely produced by “ping-pong” independent mechanism, iii) the core components of the siRNA and piRNA biogenesis were under avoidance of the negative feedback regulatory loop.

### Clustering siRNAs and piRNAs in the genome of *An. gambiae*

**Figure 5:**
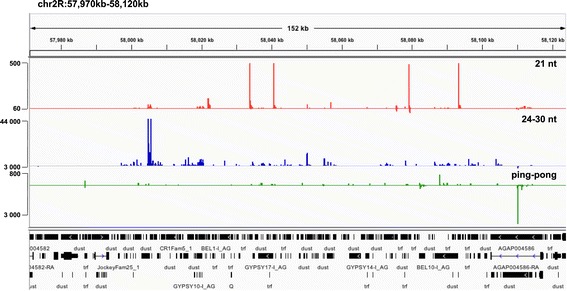
**Pattern of clustered siRNA and piRNA transcriptional activity in** ***An. gambiae*** **.** Graph showing hot spots of clustering siRNAs and piRNAs in respect to genomic distance in the *An. gambiae* genome. siRNAs (red), piRNAs (blue) and “ping-pong” piRNA pairs (green) in the clustering loci are shown as indicated*.*

In *Drosophila,* the majority of piRNAs (~92%) derive from uni- and dual-strand piRNA clusters that occupy only 3.5% of the fly genome and consist largely of transposon sequences [15]. The genomic loci producing clustering piRNAs are often associated with clustering siRNAs [40,49]. We analysed the genomic piRNA and siRNA cluster distribution in collapsed sugar-fed mosquito libraries by scoring for clustering of the uniquely and multiply mapped 21-nt reads and 24–30-nt reads (Methods; Table 2, Additional file 7: Table S5). We found that the chromosome 2 was highly enriched for total abundance of both uniquely and multiply mapped clustering piRNAs and siRNAs (Figure 5, Additional file 7: Table S5). A detailed analysis of transcriptional activity of the clustering small RNAs revealed that the most abundant piRNA and siRNA clusters were associated with TE clusters on chromosomes X and 2R, respectively (Figure 5; Additional file 7: Table S5). In *Drosophila* the most abundant piRNA cluster size ranged from 2 to 242 kb and in *Aedes* mosquitoes - from 6 to 184 kb [15,32]. In *An. gambiae* the size of the most abundant genic piRNA and TE-piRNA clusters was 170 kb and 520 kb respectively (Additional file 7: Table S5), suggesting that *An. gambiae* piRNA clusters cover greater genomic distances then in the genome of *D. melanogaster* or *Ae. aegypti*. We also noticed that piRNA clusters produced more than 90% of the detected piRNAs in *An. gambiae*; similarly to 84% of the observed piRNAs produced by the piRNA clusters in *A. agypti* [32]. In *An. gambiae,* clustering TE-piRNAs predominantly mapped to the antisense strand, whereas genic clustering piRNAs were in sense orientation (Additional file 7: Table S5). In addition, clustering siRNAs were mapped preferentially to the sense strand of both, protein coding genes and TEs in our libraries.

**Table 2:** **The top 30 abundant TE-associated piRNA clusters in** ***An. gambiae***

**chr**	**Start**	**End**	**Length**	**Unique read number**	**Total read number**	**S unique read number**	**AS unique read number**	**S total read number**	**AS total read number**
chrX	22 952 163	23 481 504	529 341	12 617	524 714	5 662	6 955	306 526	218 188
chrX	23 518 519	23 797 847	279 328	6 050	287 157	3 153	2 897	185 847	101 310
chrX	24 283 538	24 392 789	109 251	5 931	234 948	1 943	3 988	67 057	167 891
chrX	3 290 550	3 362 878	72 328	4 494	215 967	633	3 861	19 123	196 844
chrX	21 838 300	22 218 800	380 500	5 680	179 338	1 955	3 725	64 596	114 742
chrX	11 596 752	11 650 633	53 881	4 346	172 217	244	4 102	6 293	165 924
chrX	20 625 699	20 944 044	318 345	4 898	158 982	1 456	3 442	39 427	119 555
chrX	22 577 869	22 914 102	336 233	4 284	146 406	1 298	2 986	40 480	105 926
chrX	20 176 462	20 391 304	214 842	4 165	128 553	1 350	2 815	42 931	85 622
chrX	20 970 879	21 149 974	179 095	3 407	116 074	1 015	2 392	34 978	81 096
chrX	22 313 238	22 549 636	236 398	2 653	69 651	875	1 778	23 572	46 079
chrX	20 428 815	20 603 496	174 681	2 446	59 885	774	1 672	18 433	41 452
chr2L	89	481 414	481 325	7 676	208 890	2 085	5 591	58 609	150 281
chr2L	502 755	1 019 850	517 095	6 552	196 698	1 851	4 701	48 018	148 680
chr2L	5 079 181	5 342 762	263 581	3 826	140 432	932	2 894	22 110	118 322
chr2L	1 641 056	1 919 208	278 152	3 433	90 588	952	2 481	21 187	69 401
chr2L	5 363 783	5 645 438	281 655	2 881	86 167	641	2 240	13 621	72 546
chr2R	57 973 180	58 126 345	153 165	16 504	712 727	1 404	15 100	39 319	673 408
chr2R	45 825 828	46 052 229	226 401	3 625	116 909	1 554	2 071	48 943	67 966
chr2R	60 529 536	60 775 476	245 940	3 333	98 675	944	2 389	21 048	77 627
chr2R	60 857 789	61 165 860	308 071	2 595	74 290	604	1 991	14 479	59 811
chr2R	54 533 041	54 631 947	98 906	941	66 728	228	713	8 913	57 815
chr2R	59 364 672	59 452 054	87 382	2 003	64 267	920	1 083	34 913	29 354
chr3L	20 851 509	20 867 249	15 740	3 210	186 221	22	3 188	429	185 792
chr3L	904 183	1 256 919	352 736	3 945	115 876	1 341	2 604	29 346	86 530
chr3L	4 482 742	4 823 855	341 113	3 506	95 767	1 122	2 384	28 570	67 197
chr3L	1 353 818	1 548 403	194 585	2 203	75 971	1 007	1 196	33 069	42 902
chr3L	4 891 896	5 007 445	115 549	1 781	61 830	507	1 274	14 210	47 620
chr3L	4 238 059	4 454 649	216 590	1 856	61 591	699	1 157	23 730	37 861
chr3R	23 627 315	23 733 275	105 960	2 054	75 377	363	1 691	8 532	66 845
chr3R	43 053 124	43 185 525	132 401	2 040	60 619	722	1 318	19 578	41 041

S - sense orientation with respect to the genomic coordinates, AS - antisense orientation with respect to the genomic coordinates.

A number of studies in *An. gambiae* have reported that blood meal intake induces massive changes in transcript levels 3 h after feeding compared to non-blood fed mosquitoes [50]. To determine how endogenous siRNAs and piRNAs respond to blood feeding and *P. berghei* infection, we analysed small RNA expression in mosquitoes 3 h after regular and infectious blood feeding. We quantified and assessed the differential expression of 21-nt and 24-30-nt reads in our libraries using the DESeq2.6 package [51]. We detected very few siRNAs and piRNAs with statistically significant changes in their levels in *P. berghei*-infected and blood-fed mosquitoes (Additional file 8: Table S6). It has been shown that biotic stress triggered by pathogen infection can induce *de novo* production of endogenous siRNAs and piRNAs in mosquitoes [20,30,31]. In our analysis *P. berghei* infection was not associated with *de novo* production of TE-siRNAs or TE-piRNAs. However, blood feeding induced more than ten-fold changes in the expression levels of the under-represented (<10 reads) 21-nt and 24-30-nt reads mapped to DNA transposons (Additional file 8: Table S6). Additionally, we detected only a few *de novo* produced genic piRNAs and siRNAs in the presence of *P. berghei* and a few genic piRNAs were induced by regular blood feeding (Additional file 8: Table S6) that became a source of additional piRNAs in adult females. Overall, we observed mild changes in the expression levels of endogenous siRNAs and piRNAs in adult blood-fed and *P. berghei*-infected mosquitoes that may represent a general stress response to blood feeding and infection.

### Expression of the core components of the PIWI and siRNA pathways in *An. gambiae*

**Figure 6:**
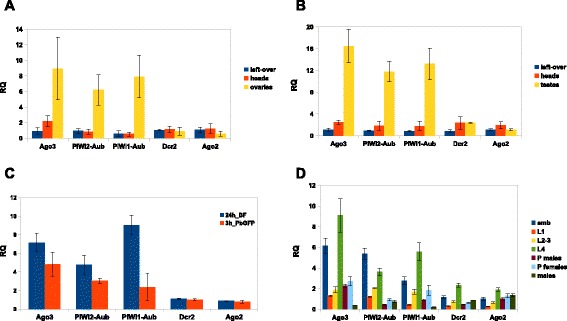
**Quantitative RT-PCR analysis of** ***Dcr-2*** **,** ***Ago-2*** **and** ***PIWI*** **-class transcripts in** ***An. gambiae*** **. (A-B)** Transcript expression profiling in adult somatic and germline organs and tissues. Transcript levels were normalized to *Rpl19* (endogenous control) and shown as a fold change above the level in one-two day old female **(A)** and male **(B)** whole body respectively. **(C)** Analysis of *Dcr-2*, *Ago-2* and *PIWI-*class transcripts 3 h after infectious feeding and 24 h after regular blood feeding in whole female body. The transcript levels are shown as a fold change above the level in sugar-fed females. **(D)** Developmental profiling showing the transcript levels in embryos (emb), 1^st^ instar larvae (L1), 2^nd-^3^rd^ instar larvae (L2-3), 4^th^ instar larvae (L4), male and female pupae (P), one-two day old male adults. Transcript levels were normalized to *Rpl19* and shown as a fold change above the level in one-two day old female whole body.

While the relevance of the piRNA and siRNA pathways in adult *An. gambiae* mosquitoes requires additional functional validation, we performed a detailed analysis of the piRNA and siRNA pathways gene expression in *An. gambiae.* We examined expression of the core components of the piRNA (*PIWI*-*Aub* and *Ago-3*) and siRNA (*Dcr-2* and *Ago-2*) pathways in adult whole body and organs composed of somatic and germline cells (Figure 6A-B). The qPCR-based measurement revealed that the *PIWI*-class transcripts were mainly expressed in adult gonads, ovaries and testes (Figure 6A-B), suggesting an essential and conserved role in germline development and maintenance. Besides, we did not observe any specific enrichment for *Dcr-2* and *Ago-2* in any of the analysed samples (Figure 6A,B,D). To determine whether the core components of the piRNA and siRNA pathways respond to blood feeding and *P. berghei* infection, we analysed the gene expression after regular and infectious feeding in mosquito females (Figure 6C; Additional file 6: Figure S3 and Additional file 9: Figure S4). The infectious blood feeding had only a mild effect on the transcript levels measured at 3 h time point (Figure 6C). Interestingly the *Dcr-2* and *Ago-2* transcript levels were specifically up-regulated in infected female guts (Additional file 6: Figure S3). In addition, we observed a significant up-regulation of the *PIWI*-class transcripts 24 h after regular blood feeding (Figure 6C).

Given the important requirement and function of the piRNA pathway during early development, we also performed a developmental profiling of the *PIWI*-class genes in *An. gambiae* (Figure 6D). There was a significant increase in the *PIWI*-class transcript expression during early stages compared with adult stage we examined. We found two peaks of the *PIWI-*class transcript expression at embryonic and L4 stages (Figure 6D); the first peak might be consistent with zygotic expression of the *PIWI*-class transcripts; the second peak was detected at the late larval stage (Figure 6D). Both peaks coincided with gonad development and formation during embryonic and larval stages in *An. gambiae*.

## Discussion

Both the siRNA and piRNA pathways are known to be involved in the control of TE activity and gene expression. Here we examined the diversity and abundance of the mixed somatic/germline piRNAs and siRNAs in *An. gambiae* adult mosquitoes. The vast majority of *An. gambiae* piRNAs and siRNAs were produced from TEs in all analysed libraries. The *An. gambiae* TE-derived siRNAs mapped predominantly to class II TEs and piRNAs mapped to class I TEs; this is consistent with the previously published observation in *Ae. aegypti* [20]. The *An. gambiae* TE-associated piRNAs showed “ping-pong” sequence signatures and were most likely produced via “ping-pong” dependent mechanism. We found that more than 90% of the TE-derived 24-30-nt reads were associated with three LTR transposons, *gypsy, copia* and *Pao-Bel* that represent the most transcriptionally active TE-piRNAs in mosquito species (this study; [20,32]). Among them, *gypsy* was the most abundant source of endogenous piRNAs that mapped predominantly to the truncated or Solo-LTR copies in the *An. gambiae* genome. Overall, our results showed that TE-piRNA production in *An. gambiae* shared the basic features of piRNA biogenesis, which is conserved among insect species [15,20,32,52]. It is worth noting that the relationship between the abundance of TE-piRNAs and piRNA-mediated silencing is complex and likely depends on both transcriptional and posttranscriptional mechanisms of silencing, which might differ among TEs [53]. Interestingly, a number of *gypsy* insertions are associated with a set of known immune genes in *An. gambiae* and plausibly influence the expression of the targeted genes [41].

piRNAs are generally produced from a long primary RNA precursors uni- and bidirectionally transcribed from piRNA clusters containing transposon sequence in random orientation [15]. Interestingly, the orientation of the primary piRNA transcript can be regulated throughout development [35]. We found that more than 90% of clustering endogenous piRNAs were produced by TEs in *An. gambiae,* similarly to *D. melagaster.* In mosquito species, large differences have been observed in genome size and content of TEs. In contrast to *An. gambiae*, *Ae. agypti* has an expansion of the piRNA biogenesis genes and a large TE load that constitutes 47% of the genome and produces only 19% of the total piRNAs [32]. Nevertheless, the TE-derived piRNAs represent an important aspect of robust control of TE activities and associated with a low rate of TE remobilization in *Ae. agypti* [32]. In *An. gambiae,* we observed a correlation between TE transcription and antisense production of piRNAs similarly to *Ae. agypti.* In addition*,* the association of clustering piRNAs with TE-derived sequences was statistically significant in *An. gambiae*, in contrast to *Ae. agypti*. Curiously, a significant proportion of the endogenous piRNA population was derived from protein coding genes in both *An. gambiae* and *Ae. agypti* species (this study, [32]) and mapped predominantly to the sense strand of host genes within gene boundaries in *An. gambiae.* We showed that a majority of the *An. gambiae* genic piRNAs were missing the ‘ping-pong” sequence signatures and most likely produced via “ping-pong” independent mechanism. Interestingly, the core components of the piRNA biogenesis were not targeted by endogenous siRNAs and piRNAs in the *An. gambiae* adult mosquitoes, unlike *Ae. agypti*. Altogether, these findings suggest that some aspects of the piRNA biogenesis and piRNA-mediated regulation might differ between distinct mosquito species.

It has been shown that in the presence of exogenous and endogenous genetic elements the production of endo-piRNAs and endo-siRNAs were induced in *Drosophila* and mosquitoes [32,49,54]. Furthermore, arbovirus infection of cells derived from natural vector *A. albopictus* and *Ae. aegypti* induced *de novo* production of virus-specific small RNAs and endogenous piRNAs mapped to TEs [9,19,20,30-32]. *An. gambiae* is a principal malaria vector in Sub-saharan Africa of a high medical importance. We found that blood feeding induced *de novo* production of genic piRNAs and piRNAs mapped to DNA transposons that became a source of additional piRNAs in adult *An. gambiae* females. However, in the presence of *P. berghei* parasite we did not detect *de novo* produced endogenous piRNAs*. An. gambiae* is also known as a primary vector of the alphavirus *O’nyong-yong*. It has been shown that antiviral defence against systemic *O’nyong-yong* infection in *An. gambiae* requires Ago-2 activity for efficient RNA silencing [8]. Yet the detailed analysis of small RNA repertoire in the infected *An. gambiae* mosquitoes using deep sequencing has been not performed so far.

Mosquito *PIWI* family members exhibit both germline specific expression restricted to gonads and somatic expression resulting in functional RNAi activity [19,20]. In our study, the core components of the siRNA and piRNA pathways were up-regulated after blood feeding in *An. gambiae* female guts and gonads respectively*.* Recently, the expression profiles of *PIWI*-class transcripts have been reported in the closely related *An. stephensi* species [55]. Both *Anopheles* species showed gonad specific enrichment for *PIWI*-class transcripts and up-regulation of their expression levels after blood feeding (this study; [55]).

TE-dependent chromosomal rearrangements are known to be involved in the shaping of genome landscapes and creating new gene blocks that influence ecological adaptation, plasticity, behaviour and vector capacity in the pan-African malaria vector, *An. gambiae* [56]. In the genome of *An. gambiae* the sex chromosome and autosomes have different genome landscapes and distinct enrichments by TEs. The distribution of TEs is higher overall near centromeres in the euchromatic regions, near the telomeres and in addition differs by chromosome arms [27]. The highest TE density is associated with the fastest evolving chromosome X, where the majority of fixed inversions were found. Importantly, the X chromosome is enriched for genes that are known to play a role in reproductive isolation and specification in *Anopheles.* A large number of autosomal paracentric polymorphic inversions has been reported on the 2R chromosome, which is associated overall with the lowest transposon and repeat densities [27]. Interestingly, the majority of the 2R arm inversions are associated with clustered segmental duplications and insertions of remnants of repetitive sequences including class I and II transposable elements [56,57]. In addition, it has been reported that the breakpoints for the chromosomal inversion 2La appear to be enriched for LTR transposons [41]. These inversions are associated with population structure, conferring ecological adaptation and altering mosquito adaptive fitness [27,58]. Further study is required to determine the precise role of the endogenous siRNAs and piRNAs in the evolution of regulatory and functional genome landscape of *An. gambiae*. Chromosomal aberration with a deleterious effect on viability and reproduction could be introduced artificially to reduce the size of the vector population, therefore representing a potential vector control strategy based on manipulation of the *An. gambiae* genome and fitness.

## Conclusion

We analysed the endogenous siRNA and piRNA populations in the African malaria mosquito *An. gambiae*. Like *Ae. agypti*, vast majority of *An. gambiae* piRNAs and siRNAs were produced by TEs from class I and class II transposons respectively. The most abundant *An. gambiae* TE-associated piRNAs were 26-27-nt in length and most likely produced by a “ping-pong” dependent mechanism*;* whereas the majority of genic piRNAs were 29-nt in length and were missing the ‘ping-pong” sequence signatures. Vast majority of the detected piRNAs were produced from TE-associated clusters in *An. gambiae,* similarly to *D. melanogaster.* Overall, TE-siRNAs and TE-piRNAs were expressed at much higher levels than genic siRNAs and piRNAs. The main components of the siRNA and piRNA biogenesis were under avoidance of being targeted by endogenous siRNAs and piRNAs in adult *An. gambiae* mosquitoes. Importantly, *An. gambiae* adult gonads were highly enriched by *PIWI*-class transcripts indicating the existence of a conserved mechanism, which controls the expression and function of *PIWI-*class genes in mosquitoes. In addition, the *PIWI*-class transcripts and two core components of the siRNA pathway, *Dcr-2* and *Ago-2* were up-regulated after regular and infectious blood feeding in the *Anopheles* female body and infected female guts respectively. Moreover, blood feeding and *P. berghei* infection induced *de novo* production of genic piRNAs that became a source of additional piRNAs in adult females. The mild changes in the expression levels of endogenous siRNAs and piRNAs were observed in response to blood feeding and *P. berghei* infection, which might represent a general response to biotic stress.

## Methods

### Mosquito rearing and sample collection

*An. gambiae* G3 strain was reared and maintained in humidified chambers at 28°C with a 12 h light/dark cycle. Mosquito samples for quantitative real-time PCR (qRT-PCR) profiling were prepared from ten-fifteen individuals. One-two days old adult whole bodies, heads, adult gonads (testes and ovaries) and leftover were collected from sugar-fed female and male mosquitoes. Mosquito feeding on anaesthetized *PbGFPCON-*infected mouse and on a regular mouse was performed as described in [33]. Three and twenty-four hours post regular and infectious blood feeding, whole bodies, heads, ovaries, guts and left-over were collected from blood-fed and *P. berghei-*infected four-five and six day old female mosquitoes respectively. Sugar-fed mosquitoes were used as a control. Developmental profiling was performed on a mixture of the fertilized eggs (0-24 hours after oviposition), a mixture of early larval (L1), intermediate (L2-3) and late larval stages (L4), female and male pupae, one-two day old adult female and male mosquitoes.

### Small RNA analysis

To identify endogenously produced siRNAs and piRNAs in *An. gambiae* adult females we used six small RNA libraries reported in ([33], GEO accession number GSE50396). The ncPRO-seq analysis pipeline was used to filter out known *rRNAs*, *tRNAs*, *snRNAs*, *snoRNAs* and *miRNAs* [59]. The remaining reads were mapped to *An. gambiae* AgamP3.8 PEST genome assembly (VectorBase) using Bowtie program allowing two mismatches. For the repetitive and mobile elements associated small RNA analysis, all TEs were retrieved from the EnsemblMetazoa database. The sense and antisense small RNA quantifications were performed with a pipeline of custom developed Python scripts. Uniquely and multiply mapped 21-nt reads and 24-30-nt reads were analysed independently and normalized by their read numbers against genomic locations. The raw and normalized read frequency of small RNA reads derived from repetitive elements and TEs or coding genes are listed in Additional file 2: Table S2, Additional file 3: Table S3, Additional file 10: Table S4 and Additional file 8: Table S6, respectively. IGV2.3.31 viewer was used to visualize sequence reads mapped to the reference genome.

The overlap pair analysis was performed using collapsed datasets obtained for sugar-fed mosquitoes allowing two mismatches. Quantification of the 21-nt and 24-30-nt paired reads was performed using a pipeline of custom developed Python scripts available upon request. The following pair arrangements between 5′ ends of the reads and their neighbour reads on the opposite strand were analysed for siRNA pairs (21-nt reads in length with the 3′-end 2-nt overhangs, offset +19-nt) and for piRNAs (24-30-nt read length; offset in 5′-nucleotides 0 nt; +10 nt or –(16-18) nt as described in [15,16]. Five reads cut-off was used for the paired sense and antisense strands mapping and the read numbers were then normalized for each data set. The piRNA cluster analysis was performed as described in [15,16]. We used a 5-kb window across the *An. gambiae* genome to identify all regions with frequencies greater than one siRNA or piRNA/kb. Only uniquely mapped 24-30-nt reads were considered for the further analysis with a cut-off of 10 reads and more (Additional file 7: Table S5). Sequence compilations for 21-nt and 24-30-nt reads based on relative nucleotide frequency per each position across the reads mapped to sense and antisense strands, separately, were generated using WebLogo3.3. For all WebLogos, libraries from two replicates obtained from sugar-fed females were collapsed. The DESeq2.6 package was used to quantify and to assess differential expression of 21-nt and 24-30-nt reads, which was considered as significant at *p < 0.05* (shown in Additional file 8: Table S6).

### Plasmid construction and RNA-based silencing

RNA interference was used to silence gene expression in adult female mosquitoes. *Ago-3* (*AGAP008862*), *PIWI2/Aub* (*AGAP009509*) and *PIWI1/Aub* (*AGAP011204*) fragments were PCR-amplified from a cDNA (*An. gambiae* G3 strain) and cloned into pLL110 vector carrying two T7 promoters described in [60]. *Dcr-2* (*AGAP012289*): *EcoRI-HindIII* 512-bp fragment was subcloned from the 71B02 clone of the Gateway (Invitrogen) immune library described in [61] into the pLL110. The pLL110-*Ago-2* previously described in [33]. The following PCR primers were used, *Ago-3* forward *5′-GTCAAACATGTACCGCCGTGTG-3′*; *Ago-3* reverse primer: *5′-CCCCATGATCTGTGGCATTGAC-3′*; *PIWI2/Aub* forward primer: *5′-GCAAACCTCCCCCGAAAGCC-3′*; *PIWI2/Aub* reverse primer *5′-CGACACCACGCACATGATCATC-3′*; *PIWI1/Aub* forward primer: *5′-GCGACAAGTCGCTCTCGTACGG-3′*, *PIWI1/Aub* reverse primer: *5′- GTACTGGCAGACAGCCGGTAC-3′*. Double-stranded RNAs were prepared as described in [60]. One-day post-emerged CO_2_-anaesthetized mosquito females were injected intrathoracically with 0.6 μg of dsRNA using nano-injector (Nanoject II, Drummond). dsRNA of the *lacZ* gene was used as a control. Efficacy of RNA silencing on gene expression was analysed 24 h after dsRNA injection by qRT-PCR.

### qRT-PCR

Gene expression profiling and the efficacy of RNA silencing were assessed by Fast SYBR Green-based qPCR (ABI). Total RNA was isolated using a TRI Reagent (MRC). cDNAs were synthesized from 0.4 μg of total RNA samples using random hexamer primers and Revert Aid H Minus cDNA synthesis kit (Thermo Scientific). For the PCR amplification of *gypsy* and *CR1* transcripts, total RNA samples (0.4 μg) were pre-treated with DNAse I enzyme (Thermo Scientific). After inactivation of the enzyme, the RNA samples were used for cDNA synthesis as described above; no reverse transcriptase controls were used to access for genomic DNA contamination in the DNAse I pre-treated RNA samples. qPCR was performed using the following primers: *Dcr-2* forward primer: *5′-GCGAAGGCCAGGTAATTATCTG-3′*, *Dcr-2* reverse primer: *5′-GACATTCGTCGAACACGATCA-3′*; *Ago-2* forward primer *5′-ATGCTCAAGATCAACGCCAAA-3′*, *Ago-2* reverse primer *5′-TGAGCGGGTGCGTAACGT-3′*; *Ribosomal protein L19* (*RpL19)* forward primer *5′-CCAACTCGCGACAAAACATTC-3′*, *RpL19* reverse primer *5′-ACCGGCTTCTTGATGATCAGA-3′*; *Ago-3* forward primer *5′-CATAAGGTAATGCGCGATATTGC-3′*, *Ago-3* reverse primer *5′-CGGCTTCATTTTTGTTCACATTC-3′*; *PIWI2/Aub* forward primer: *5′-GCGCTCCGATTTCAAAATGA-3′*, *PIWI2/Aub* reverse primer *5′-CGTTTCCAGCCGTTCGATA-3′*; *PIWI1/Aub* forward primer: *5′- GCCGCAGATCCTTTATTTGC-3′*, *PIWI1/Aub* reverse primer: *5′*-*GGTCGGCCCGATCGTT-3′*; *AGAP003387* forward primer: *5′-AAATCAGTGCGTGTGTCCGAAC-3′*, reverse primer: *5′- GCGATGGGTCGTTTCCTTACGGTG-3′*; *AGAP001052* forward primer: *5′-CCACTCAAGTTTATGTGGTCTATGGA-3′*, reverse primer: *5′-TCAGATGATGATTGACCTCGTAGAA-3′; gypsy (GYPSY10-I_AG)* forward primer: *5′-CCAGATGACTCGAAATACGATAGC-3′*, *gypsy (GYPSY10-I_AG)* reverse primer: *5′-GTTTAGCGGTTTTGCTTTCAAAG-3′*; *CR1-9_AG* forward primer: *5′-TCGACTCCTTCCATGGCAAT-3′, CR1-9_AG* reverse primer: *5′-*AAGCAGACACCGCTGATGGT*-3′. RplL19* was used as an endogenous control to normalize the transcript levels. PCR reactions were performed on an OneStep Plus thermocycler (ABI), according to the manufacturer’s protocol. Each measurement was derived from three independent biological replicates for all expression profile analyses performed in this study. Relative quantification of gene expression was performed using the comparative Ct (ΔΔCt) method.

### Ethics statement

All experimental procedures on mice were performed in accordance with the national animal protection law (Landesamt für Gesundheit und Soziales (LAGeSo)) and approved by the committee for animal use and protection (LAGeSo permit number: H 0027/12).

## Additional files

Additional file 1: Table S1. Small RNA size distribution and abundance in *An. gambiae* libraries. Additional file 2: Table S2. Abundance of small RNAs from *An. gambiae* libraries mapped to TEs. Additional file 3: Table S3. Abundance of small RNAs from *An. gambiae* libraries mapped to *An. gambiae* coding genes. Additional file 4: Figure S1. Frequency and distribution of 21-nt and 24-30-nt reads mapped to the most abundant NLTR-transposons, DNA transposons and specific coding genes. Additional file 5: Figure S2. qPCR-based measurement of the transcript levels (the most representative TEs and coding genes) in *Dcr-2*, *Ago-2* and *PIWI* silenced mosquitoes. Additional file 6: Figure S3. Expression profiling of the core components of the siRNA and piRNA pathways and the most representative coding genes after regular and infectious blood feeding in *An. gambiae* females. Additional file 7: Table S5. The siRNA and piRNA cluster analysis in the *genome of An. gambiae*. Additional file 8: Table S6. Differential expression analysis of siRNAs (21-nt) and piRNAs (24-30-nt) in *Anopheles* females 3 h after regular and infectious blood feeding. Fold-changes in the expression levels are shown as a ratio of blood-fed (BF) to sugar-fed (SF) mosquitoes or as a ratio of infected (*PbGFP*) to non-infected blood-fed mosquitoes (BF). Additional file 9: Figure S4. Expression profiling of the core components of the siRNA and piRNA pathways in sugar and blood-fed *An. gambiae* females. Additional file 10: Table S4. The top abundant genes producing endogenous siRNAs and piRNAs in *An. gambiae* libraries (two collapsed replicate).

## Abbreviations

siRNA: small interfering RNA
piRNA: PIWI-interacting RNA
TE: Transposable element
ncRNA: non-coding RNA
RISC: RNA-induced silencing complex
Aub: Aubergine
PIWI: P-element induced wiped testis
Ago: Argounate
Dcr: Dicer
LTR: Long terminal repeat
NLTR: Non-long terminal repeat
MITE: Miniature inverted transposable element
SINE: Short interspersed nuclear element
*cis*-NAT: *cis*-natural antisense transcript
nt: Nucleotide
kb: Kilobase
h: Hour
