# Supplementary material for: Endogenous siRNAs and piRNAs derived from transposable elements and genes in the malaria vector mosquito Anopheles gambiae
Source: BMC Genomics. 2015 Apr 10;16(1):278. doi: 10.1186/s12864-015-1436-1 (PMC4423592; doi:10.1186/s12864-015-1436-1)
Supplement: Additional file 4: Figure S1. — Frequency and distribution of 21-nt and 24-30-nt reads mapped to the most abundant NLTR-transposons, DNA transposons and specific coding genes. [file 12864_2015_1436_MOESM4_ESM.pdf]

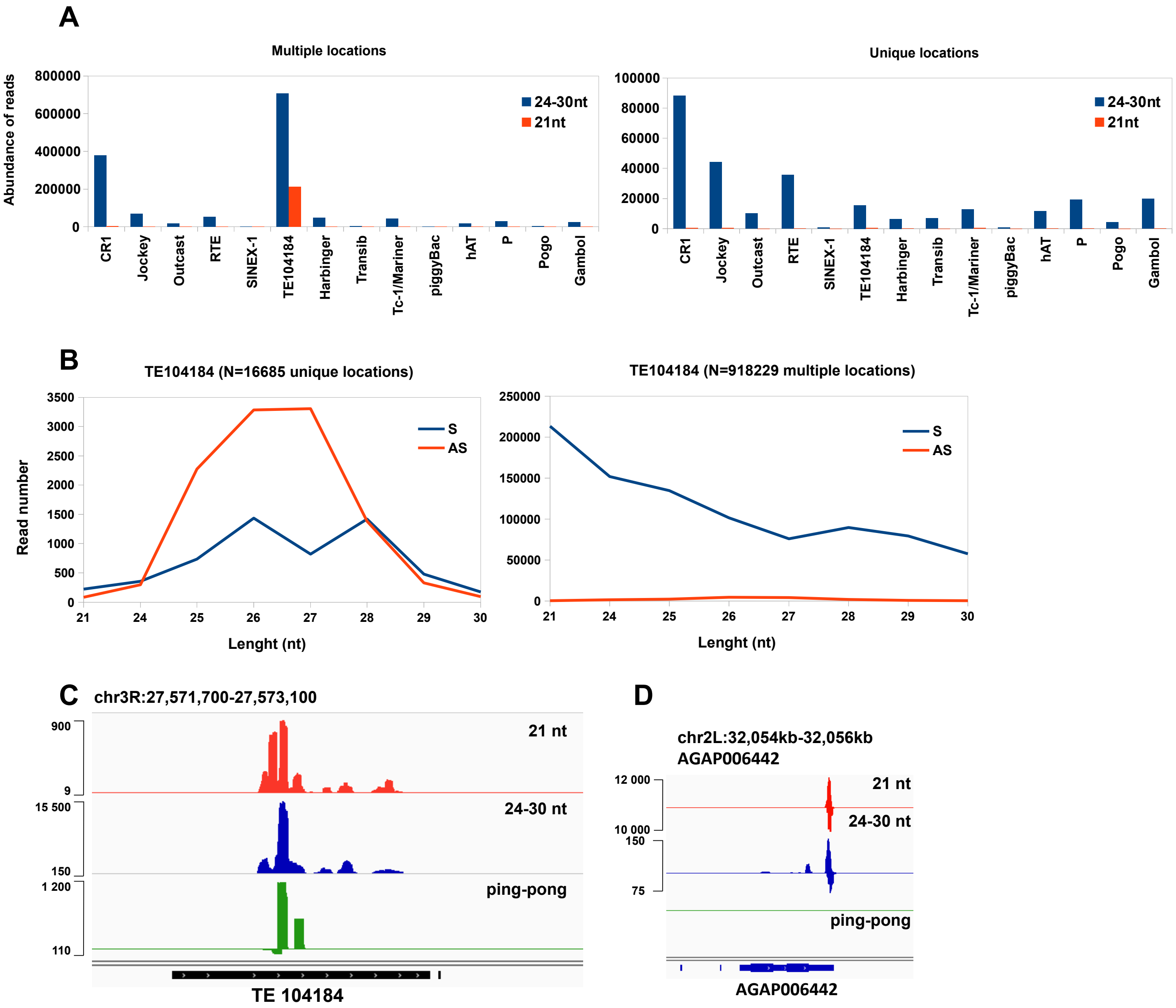

Additional file 4. Figure S1

(A) Read frequency for siRNAs (21 nt) and piRNAs (24-30 nt) mapped to the most abundant NLTR and DNA transposons. (B) Read frequency and distribution mapped to the unknown *TE element 104184* (class II DNA transposons) uniquely mapped to *An. gambiae* genome (left) and multiply mapped reads to the genome (right). (C-D) Density and distribution of small RNAs mapped to *TE104184* transposon (C) and *AGAP006442* (D); siRNAs (red), piRNAs (blue) and piRNA ping-pong pairs (green) are as indicated.
