# Supplementary material for: Endogenous siRNAs and piRNAs derived from transposable elements and genes in the malaria vector mosquito Anopheles gambiae
Source: BMC Genomics. 2015 Apr 10;16(1):278. doi: 10.1186/s12864-015-1436-1 (PMC4423592; doi:10.1186/s12864-015-1436-1)
Supplement: Additional file 5: Figure S2. — qPCR-based measurement of the transcript levels (the most representative TEs and coding genes) in Dcr-2, Ago-2 and PIWI silenced mosquitoes. [file 12864_2015_1436_MOESM5_ESM.pdf]

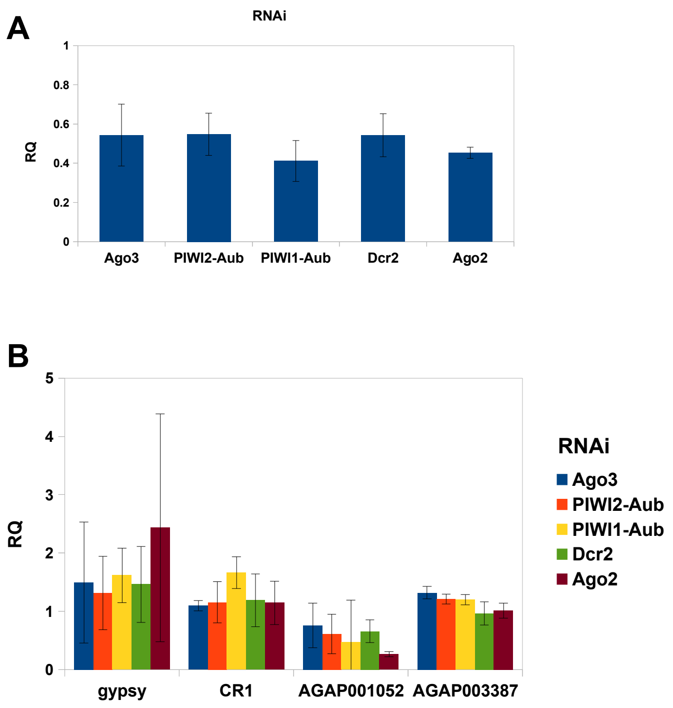

Additional file 5. Figure S2

(A) Gene silencing of *PIWI-class, Dcr-2* and *Ago-2* transcripts in *An. gambiae* females 24 h after dsRNA injection measured by qRT-PCR. In all graphs, the transcript levels were normalized to *Rpl19* and shown as a fold change relative to the level in *lacZ* control. (B) qRT-PCR analysis of *gypsy* and *CR1* transcripts (the most representative LTR- and NLTR-retrotransposons, respectively), *AGAP003387* and *AGAP001052* expression in *Dcr-2*, *Ago-2* and *PIWI*-class silenced mosquitoes.
