# Supplementary material for: Endogenous siRNAs and piRNAs derived from transposable elements and genes in the malaria vector mosquito Anopheles gambiae
Source: BMC Genomics. 2015 Apr 10;16(1):278. doi: 10.1186/s12864-015-1436-1 (PMC4423592; doi:10.1186/s12864-015-1436-1)
Supplement: Additional file 6: Figure S3. — Expression profiling of the core components of the siRNA and piRNA pathways and the most representative coding genes after regular and infectious blood feeding in An. gambiae females. [file 12864_2015_1436_MOESM6_ESM.pdf]

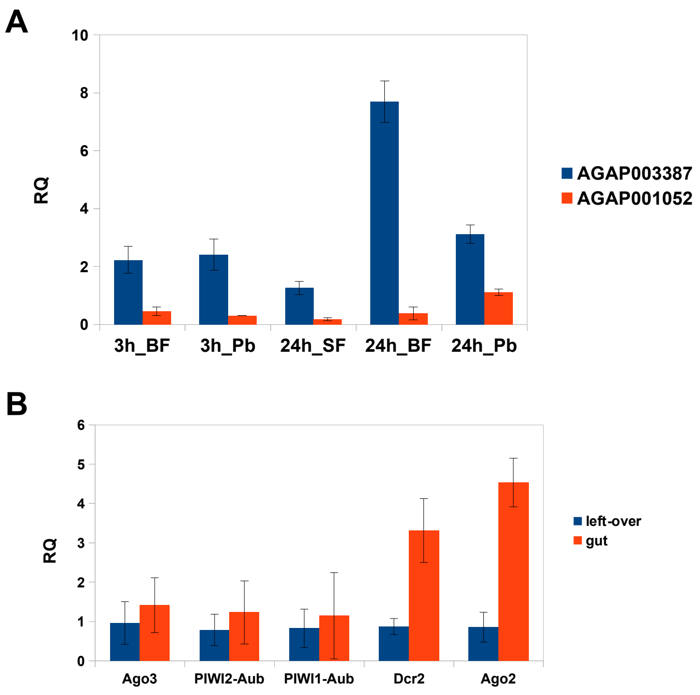

Additional file 6. Figure S3

(A) Quantitative RT-PCR analysis of *AGAP003387* and *AGAP001052* transcripts 3h and 24 h after regular blood feeding and after *PbGFP* infection in *An. gambiae* females. (B) Expression of *Dcr-2*, *Ago-2* and *PIWI*-class transcripts in four-five day old females 3h after *PbGFP* infection. The transcript levels were measured by qRT-PCR and shown as a fold change above the level respectively in sugar-fed (A) and *PbGFP*-infected female whole body (B).
