# Supplementary material for: Endogenous siRNAs and piRNAs derived from transposable elements and genes in the malaria vector mosquito Anopheles gambiae
Source: BMC Genomics. 2015 Apr 10;16(1):278. doi: 10.1186/s12864-015-1436-1 (PMC4423592; doi:10.1186/s12864-015-1436-1)
Supplement: Additional file 9: Figure S4. — Expression profiling of the core components of the siRNA and piRNA pathways in sugar and blood-fed An. gambiae females. [file 12864_2015_1436_MOESM9_ESM.pdf]

A

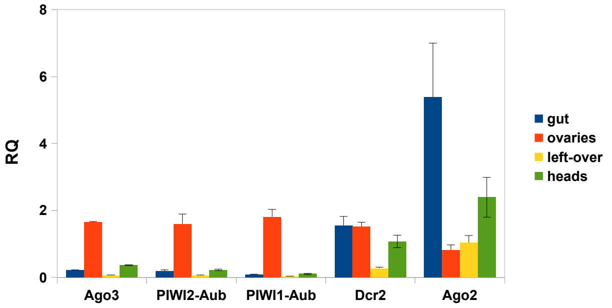

B

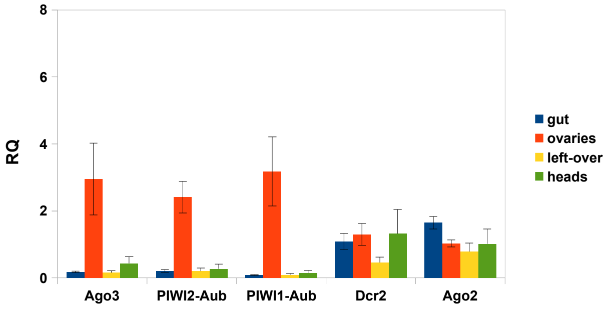

Additional file 9. Figure S4

(A-B) Quantitative RT-PCR analysis of *Dcr-2*, *Ago-2* and *PIWI*-class transcripts in five-six day old females 24 h after blood feeding females (A) and sugar-fed females (B). The transcript levels were normalized to *Rpl19* and shown as a fold change above the level in the respective female whole body.
